# Impact of frequent emergency department visits on resource utilization

**DOI:** 10.1186/s12873-026-01784-5

**Published:** 2026-10-12

**Authors:** Raif Kaan Bas, Sumeyye Cakmak

**Affiliations:** 1 Department of Medical Pharmacology, Faculty of Medicine, Research Institute for Health Sciences and Technologies (SABITA), Istanbul Medipol University, Göztepe Mah, Kavacık, Atatürk Cd. No:40, Beykoz, Istanbul, 34810 Türkiye; 2 Department of Emergency Medicine, Istanbul Cam and Sakura City Research and Training Hospital, University of Health Sciences, Istanbul, Türkiye

**Keywords:** Emergency service, Hospital, Health resources, Health care costs, Facilities and services utilization, Patient acceptance of health care

## Abstract

**Background:**

Emergency department (ED) utilization continues to rise globally, and a disproportionate share of visits is attributable to a small subgroup of patients. This subgroup is referred to throughout the present report by the single term high-frequency users (HFUs), defined as patients with four or more ED visits within a 12-month period. Characterizing the clinical profile, utilization patterns and cost structure of HFUs is relevant to emergency care planning and resource allocation.

**Methods:**

This retrospective, single-center observational study analyzed all adult ED presentations at a high-volume urban tertiary hospital over a 12-month period. HFU status was defined at the patient level as four or more ED visits within the study period. The unit of analysis was specified for every comparison: age, sex, the ICD-10 diagnostic spectrum and all logistic regression models were analyzed at the patient level (one observation per unique patient), whereas triage category, timing of presentation, mode of arrival, resource utilization and per-visit costs were analyzed at the visit level. For the regression models, visit attributes and diagnostic categories were aggregated to the patient level as ever/history indicators. Groups were compared using the Mann–Whitney U and Pearson chi-square tests, with effect sizes reported alongside *p* values.

**Results:**

Among 466,884 unique adult patients accounting for 726,696 ED presentations, 26,871 patients (5.8%) were HFUs. HFUs represented 5.8% of adult patients but accounted for 148,322 of 726,696 presentations (20.4%) and approximately one-fifth (21.0%) of cumulative laboratory, imaging and medication costs. Total cost per visit was similar between groups (median 6.39 USD in both; *p* = 0.647), and differences in individual cost components, although statistically significant, were small in practical terms (rank-biserial correlations +0.03 for laboratory, −0.05 for radiology and +0.08 for medication cost). A higher proportion of HFU than non-HFU presentations involved any laboratory test (29.3% vs 26.7%) and any medication order (57.8% vs 51.4%), whereas any radiologic imaging was less frequent among HFUs (40.5% vs 44.7%). HFUs were marginally younger, presented more often during the night shift and were less likely to arrive by ambulance (3.5% vs 5.0%). Yellow-triage presentations predominated among HFUs, and symptom-based and pain-related diagnoses were over-represented. In the patient-level multivariable model, male sex, a history of yellow- or red-triage presentation, a history of evening- or night-shift presentation and multiple symptom-based diagnostic groups were associated with HFU status, whereas a history of ambulance arrival was associated with lower odds.

**Conclusions:**

HFUs constitute a small proportion of adult ED patients but account for a disproportionate share of presentations and of cumulative diagnostic and pharmacologic resource use. Because total cost per visit was similar between the groups and the differences in individual cost components were small in practical terms, the principal finding is the cumulative resource burden associated with repeated attendance rather than a higher cost of the individual encounter. This pattern is consistent with a contribution of out-of-hours access and symptom-driven presentation; however, the present observational data cannot establish these mechanisms or identify which interventions would reduce frequent ED use.

## Introduction

Emergency department (ED) utilization has increased steadily worldwide, placing growing pressure on emergency care systems. National analyses from the United States demonstrate a sustained rise in adult ED visits over time, reflecting an expanding reliance on emergency services across diverse patient populations [1].

In parallel with rising utilization, the financial burden of ED care has escalated substantially. Emergency services represent one of the fastest-growing components of healthcare spending, with U.S. data showing a marked increase in ED treatment costs in recent years, attributable largely to higher costs per visit rather than to patient volume alone [2].

A consistent finding across the literature is that a disproportionate share of ED utilization is attributable to a relatively small subgroup of patients. Such patients—commonly defined as individuals with multiple ED visits per year—constitute a minority of patients yet account for a substantial proportion of total ED visits and resource consumption [3, 4]. For clarity and consistency, the single term high-frequency user (HFU) is used throughout this report for this group, including where the cited literature uses alternative labels such as “frequent user” or “super-utilizer”. This pattern has been observed across diverse populations, including pediatric patients and individuals with substance use disorders, suggesting that high-frequency ED use is not limited to a single demographic or clinical group [5, 6]. Population-based analyses further confirm that HFUs contribute disproportionately to ED crowding and healthcare costs [7].

Despite extensive characterization of HFUs, important gaps remain. Most existing evidence originates from North American and Western European health systems, and comparatively few studies have examined high-frequency use within publicly funded systems that permit unrestricted, self-referred and essentially cost-free access to emergency care, such as the Turkish health system [8–10]. Moreover, prior work has largely reported aggregate utilization or total cost [2, 11], whereas the specific cost components associated with resource consumption—laboratory testing, radiologic imaging and pharmacologic treatment—have rarely been disaggregated and linked to the underlying diagnostic spectrum. This distinction matters because total cost per visit may obscure clinically meaningful differences in how resources are consumed.

This study addresses these gaps using one of the largest single-center ED cohorts examined for this purpose—726,696 adult presentations from 466,884 patients at a high-volume urban tertiary hospital in Türkiye. We jointly characterize the demographic profile, triage acuity, temporal patterns and full ICD-10 diagnostic spectrum of HFUs, while disaggregating resource utilization into laboratory, imaging and medication cost components and distinguishing throughout between patient-level and visit-level findings. By situating these findings within a universal-coverage system with open ED access, the study aims to clarify what distinguishes HFUs beyond prevalence alone and to identify the diagnostic and cost domains most relevant to emergency care planning.

## Methods

### Study design

This study was a retrospective, cross-sectional observational cohort analysis of adult emergency department visits conducted over a 12-month period (1 July 2024–30 June 2025) at a single high-volume urban tertiary hospital. The study protocol was approved by the Clinical Research Ethics Committee No. 1 of Başakşehir Çam and Sakura City Hospital (protocol/decision number 2025–262; date of approval 27 August 2025) and the study was conducted in accordance with the Declaration of Helsinki.

### Study population, data source and exclusions

All ED visits by patients aged ≥ 18 years during the study period were eligible for inclusion. Records were extracted from the hospital information management system. The raw extract comprised 789,690 ED presentation records. Of these, 62,994 records (8.0%) were excluded for the following reasons: age under 18 years; a missing or invalid national identity number, which precluded reliable linkage of visits to a unique patient; duplicate transaction entries for the same presentation; technical registration errors; and cancelled or voided presentations. Records were additionally classified as incomplete, and excluded, if any of the following key variables was missing: the anonymised unique patient identifier, age, sex, date and time of presentation, or triage category. Counts for the individual exclusion reasons were not retained separately during data cleaning. The final analytic dataset comprised 466,884 unique adult patients accounting for 726,696 ED visits.

Patients were categorized according to their cumulative number of ED visits during the study period. HFUs were defined as patients with four or more ED visits within the 12-month period; patients with fewer than four ED visits were classified as non-HFUs. The threshold of four or more visits per year was selected a priori, as it is the most commonly applied definition of frequent ED use and the level at which, in population-based data, a small subgroup begins to account for a disproportionate share of ED visits, thereby maximizing comparability with prior studies [8, 9, 12]. No universal definition exists; some authors have adopted higher thresholds to delineate “highly frequent” users or “super-utilizers” (e.g. ≥7 or ≥ 10 visits per year) [11, 13]. We retained the ≥4 definition to balance identification of a clinically meaningful high-utilization subgroup against the need to preserve an adequate subgroup size.

### Variables and definitions

Variables comprised age, sex, triage category, diagnostic patterns, mode of arrival (ambulance vs. self-presentation), timing of ED visits and healthcare resource utilization. HFU status was defined at the patient level on the basis of annual ED visit frequency.

Triage categorization was performed using the three-tier color-coded system applied nationally in Türkiye, which integrates key components of the Emergency Severity Index (ESI) framework. Red (immediate/critical) denotes patients with an actual or imminent threat to life or a vital-organ function who require resuscitation or immediate intervention, corresponding to ESI levels 1–2. Yellow (urgent) denotes patients who are hemodynamically stable at presentation but whose complaint requires prompt physician assessment, observation, or diagnostic work-up because of the potential for deterioration, corresponding broadly to ESI level 3. Green (non-urgent) denotes ambulatory patients with minor or self-limiting complaints who can safely wait and are typically managed without extensive resource use, corresponding broadly to ESI levels 4–5. The triage category was assigned at the point of registration by a trained triage nurse and was subsequently verified, and where necessary revised, by the attending emergency physician at first clinical contact; the category recorded in the hospital information system after this verification was the one used for analysis.

The emergency department operates on a three-shift system, each lasting eight hours: morning (08:00–16:00), evening (16:00–24:00) and night (24:00–08:00). Presentations occurring during the evening and night shifts were considered out-of-hours attendances. Diagnoses were recorded using ICD-10 codes and were analyzed at the level of the three-character root code.

### Resource utilization and cost analysis

Healthcare resource utilization was assessed using laboratory testing, radiologic imaging and pharmacologic treatments administered during ED visits. For each of these three components, both the proportion of presentations in which the resource was used at all and the associated cost were examined. Cost data were extracted from the institutional billing system, which records charges according to the national Social Security Institution reimbursement tariff (Health Implementation Communiqué; Sağlık Uygulama Tebliği, SUT), and were converted to U.S. dollars (USD) at a fixed exchange rate of 35 Turkish lira per USD applied at the time of analysis. The resulting per-visit values are accordingly low, as they reflect reimbursed national-tariff prices rather than commercial or list charges.

Total visit cost was defined as the sum of all charges billed for a single ED presentation. In addition to the three components analyzed separately (laboratory testing, radiologic imaging and medication orders), it includes the ED examination (physician encounter) fee, bedside and therapeutic procedures, consumables and materials, observation charges and any consultation fees generated during the presentation. Total visit cost therefore exceeds the sum of the three reported components. Costs incurred after ED disposition—including inpatient care following admission, outpatient follow-up, and indirect or societal costs—were not captured. Costs were analyzed at the visit level and, in addition, summed across all of a patient’s visits to yield cumulative annual per-patient costs for the patient-level comparison.

### Unit of analysis

The unit of analysis was specified explicitly for each comparison and is stated in every table title and figure caption together with the corresponding denominator. Patient-level analyses (one record per unique patient; *n* = 466,884) comprised age and sex, the diagnostic spectrum (the proportion of patients in whom each ICD-10 category was ever recorded during the study period), the cumulative annual per-patient costs, and all univariable and multivariable logistic regression models. Visit-level analyses (all presentations; *n* = 726,696) comprised triage category, shift of presentation, mode of arrival, resource-utilization rates and per-visit costs, and characterize the nature of the presentations rather than of the patients.

For the regression models, visit-level attributes were aggregated to the patient level as ever/history indicators: a patient was coded as having a history of yellow- or red-triage presentation, of evening- or night-shift presentation, and of ambulance arrival if the characteristic was present at any visit during the study period, and each diagnostic group was coded as ever versus never recorded. This aggregation ensured that every regression covariate was expressed at the patient level, aligned with the patient-level definition of the outcome (HFU status). It also has an important interpretive consequence, since patients with a greater number of presentations necessarily had more opportunities to satisfy any given ever/history condition; the resulting estimates are therefore reported as factors associated with HFU status and are not to be read as causal or validated predictive effects.

### Statistical analysis

Statistical analyses were performed using Python (version 3.9). Continuous variables were assessed for normality using visual inspection of histograms and Q–Q plots. As age and cost variables demonstrated non-normal (right-skewed) distributions, they were summarized as medians with interquartile ranges (IQRs) and compared using the Mann–Whitney U test. Categorical variables were reported as frequencies and percentages and compared using the Pearson chi-square test. For the cost comparisons, effect sizes—the rank-biserial correlation (Cliff’s delta) and the common-language effect size—were additionally calculated to distinguish statistical from practical significance, given the large sample size.

To identify factors associated with high-frequency ED use, binary logistic regression analyses were conducted at the patient level (one observation per patient). Both univariable and multivariable models were constructed, with the multivariable model adjusted for age, sex and the clinical covariates described in section “Unit of analysis”. Results were reported as odds ratios (ORs) and adjusted odds ratios (aORs) with 95% confidence intervals (CIs). A two-sided *p* value < 0.05 was considered statistically significant. The diagnosis Z20 (contact with and suspected exposure to communicable diseases) was excluded from both the univariable and the multivariable regression models, as its implausibly large adjusted estimate reflected its use as an administrative, screening-related code rather than a clinical correlate of high-frequency use; it is retained in the descriptive diagnostic table only.

The discrimination of the multivariable model was quantified using the area under the receiver operating characteristic (ROC) curve (AUC). This value represents the apparent discrimination of the model within the dataset in which it was fitted. Neither internal validation by resampling (e.g. bootstrapping or cross-validation) nor external validation in an independent cohort was performed; the reported AUC is therefore likely to be optimistic and should not be interpreted as evidence of validated predictive performance.

Because each patient contributed a single observation to the regression models, these inferential analyses satisfy the independence-of-observations assumption, and clustering or generalized estimating equation approaches were therefore not required. For the visit-level descriptive comparisons, repeated presentations from the same patient are not statistically independent; given the very large number of visits, at which statistical significance is readily attained, these comparisons were interpreted together with absolute differences and effect-size measures rather than with *p* values alone.

## Results

### Study population and overall emergency department utilization

**Fig. 1:**
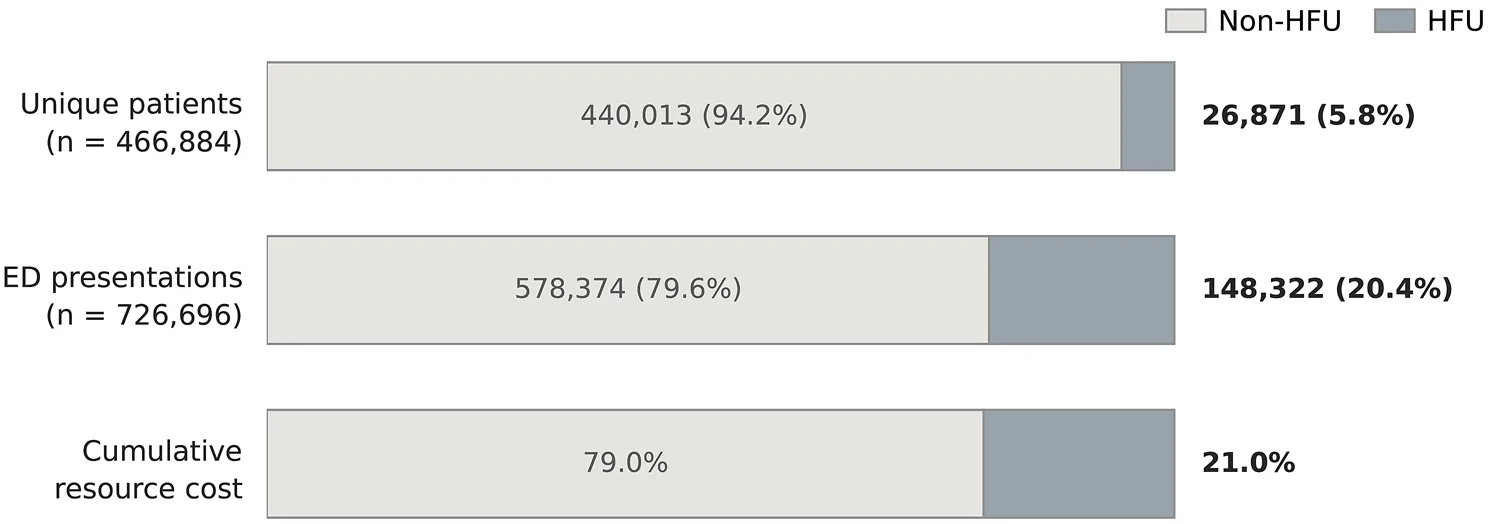
Distribution of patients, emergency department presentations and cumulative resource costs by high-frequency user status. Denominators differ by unit of analysis: unique adult patients (*n* = 466,884; HFU 26,871 [5.8%], non-HFU 440,013 [94.2%]); ED presentations (*n* = 726,696; HFU 148,322 [20.4%], non-HFU 578,374 [79.6%]); and cumulative annual laboratory, imaging and medication costs (HFU 21.0%, non-HFU 79.0%). HFU, high-frequency user (≥4 ED visits within a 12-month period)

Of 789,690 ED presentation records in the raw extract, 62,994 (8.0%) were excluded, leaving a final analytic cohort of 466,884 unique adult patients accounting for a total of 726,696 emergency department (ED) presentations during the study period. Based on annual ED utilization, 26,871 patients (5.8%) met the criteria for high-frequency use, while 440,013 patients (94.2%) were classified as non-HFUs. These 26,871 HFUs accounted for 148,322 of the 726,696 presentations (20.4%), whereas non-HFUs accounted for 578,374 presentations (79.6%) (Fig. 1).

### Demographic characteristics (patient level)

The median age was slightly lower among HFUs compared with non-HFUs (37 years [IQR 26–51] vs. 39 years [IQR 27–52]; *p* < 0.001). Sex distribution differed significantly between groups (*p* < 0.001). Females accounted for 50.9% of HFUs and 49.6% of non-HFUs, whereas males comprised 49.1% and 50.4%, respectively (Table 1). These comparisons are based on the 466,884 unique adult patients (26,871 HFUs and 440,013 non-HFUs).

**Table 1:** Demographic characteristics of unique adult patients (patient level; *n* = 466,884) and characteristics of all emergency department presentations (visit level; *n* = 726,696), by high-frequency user status

Variable	All (n = 466,884 patients; 726,696 visits) †	Non-HFU (n = 440,013 patients; 578,374 visits) †	HFU (n = 26,871 patients; 148,322 visits) †	*P***value**
**Number of patients (n)**	466884	440013	26871	-
**DEMOGRAPHIC CHARACTERISTICS**				
Age, median (IQR)	39.00 (27.00 - 52.00)	39.00 (27.00 - 52.00)	37.00 (26.00 - 51.00)	<0.001
**Sex**				<0.001
Male	235125 (50.4%)	221929 (50.4%)	13196 (49.1%)	
Female	231759 (49.6%)	218084 (49.6%)	13675 (50.9%)	
**VISIT-BASED CHARACTERISTICS**				
**Triage category**				<0.001
Red (Critical)	39155 (5.4%)	31232 (5.4%)	7923 (5.3%)	
Yellow (Urgent)	305109 (42.0%)	235963 (40.8%)	69146 (46.6%)	
Green (Non-urgent)	382432 (52.6%)	311179 (53.8%)	71253 (48.0%)	
**Shift of presentation**				<0.001
Evening	310259 (42.7%)	249315 (43.1%)	60944 (41.1%)	
Night	103923 (14.3%)	79618 (13.8%)	24305 (16.4%)	
Daytime	312514 (43.0%)	249441 (43.1%)	63073 (42.5%)	
**Arrival by ambulance**				<0.001
Yes	33850 (4.7%)	28693 (5.0%)	5157 (3.5%)	
No	692846 (95.3%)	549681 (95.0%)	143165 (96.5%)	

Notes: Values are reported as median (IQR) or number (percentage), as appropriate. Between-group comparisons were conducted using the Mann–Whitney U test for continuous variables and the chi-square test for categorical variables. A two-sided *p* value < 0.05 indicated statistical significance

Abbreviations: HFU, high-frequency user (≥4 ED visits within a 12-month period); IQR, interquartile range

†Demographic variables (age, sex) are reported per unique patient. Visit-based characteristics (triage category, shift of presentation, arrival by ambulance) are reported per ED presentation; accordingly, percentages for these rows are calculated against the total number of visits (726,696; 578,374; and 148,322 for the total, non-HFU, and HFU groups, respectively), not the number of unique patients

### Clinical characteristics of presentations (visit level)

The following comparisons are based on all 726,696 presentations (148,322 by HFUs and 578,374 by non-HFUs). Triage category distribution differed significantly between groups (*p* < 0.001). HFU presentations included a higher proportion of yellow-triage attendances than non-HFU presentations (46.6% vs. 40.8%), while green-triage attendances were less frequent among HFU presentations (48.0% vs. 53.8%). The proportion of red-triage presentations was similar between groups (5.3% vs. 5.4%).

The temporal distribution of ED presentations also differed significantly (*p* < 0.001). Night-shift presentations were more frequent among HFUs (16.4% vs. 13.8%), whereas evening-shift presentations were less common (41.1% vs. 43.1%). Day-shift presentation rates were comparable between groups.

Ambulance arrival rates differed significantly between groups, with a lower proportion observed among HFU presentations compared with non-HFU presentations (3.5% vs. 5.0%; *p* < 0.001) (Table 1).

### Diagnostic spectrum (patient level)

The distribution of the top 25 ICD-10 diagnostic categories differed significantly between HFUs and non-HFUs (all *p* < 0.001). Values represent the proportion of unique patients in whom each category was ever recorded during the 12-month study period, so that a patient may contribute to more than one category. The most frequent diagnoses among HFUs included M79 (soft tissue disorders/myalgia), J06 (acute upper respiratory infections), R52 (pain, unspecified), J39 (other upper respiratory tract diseases) and K52 (noninfective gastroenteritis and colitis). For all top 25 diagnostic categories, the proportion of affected patients was higher in the HFU group than in the non-HFU group (Table 2).

**Table 2:** ICD-10 diagnostic categories ever recorded during the 12-month study period (patient level; *n* = 466,884 unique adult patients) and costs per emergency department presentation (visit level; *n* = 726,696 presentations), by high-frequency user status

Characteristic	All (n = 466,884 patients; 726,696 visits) †	Non-HFU (n = 440,013 patients; 578,374 visits) †	HFU (n = 26,871 patients; 148,322 visits) †	*P***value**
**Diagnosis Groups (ICD-10 Codes)**				
Other Soft Tissue Disorders (M79)	241762 (51.8%)	218011 (49.5%)	23751 (88.4%)	<0.001
Acute Upper Respiratory Infections (J06)	91745 (19.7%)	78934 (17.9%)	12811 (47.7%)	<0.001
Pain, Unspecified (R52)	84878 (18.2%)	71731 (16.3%)	13147 (48.9%)	<0.001
Other Diseases of Upper Respiratory Tract (J39)	61276 (13.1%)	51659 (11.7%)	9617 (35.8%)	<0.001
Noninfective Gastroenteritis and Colitis (K52)	23327 (5.0%)	19404 (4.4%)	3923 (14.6%)	<0.001
Abdominal and Pelvic Pain (R10)	18631 (4.0%)	15197 (3.5%)	3434 (12.8%)	<0.001
Malaise and Fatigue (R53)	18090 (3.9%)	14382 (3.3%)	3708 (13.8%)	<0.001
General Examination Without Complaint (Z00)	12436 (2.7%)	9853 (2.2%)	2583 (9.6%)	<0.001
Cystitis (N30)	11376 (2.4%)	9166 (2.1%)	2210 (8.2%)	<0.001
Nausea and Vomiting (R11)	11222 (2.4%)	8988 (2.0%)	2234 (8.3%)	<0.001
Contact With and Exposure to Infectious Disease (Z20)	3096 (0.7%)	1770 (0.4%)	1326 (4.9%)	<0.001
Headache (R51)	7200 (1.5%)	5795 (1.3%)	1405 (5.2%)	<0.001
Pain in Throat and Chest (R07)	6977 (1.5%)	5853 (1.3%)	1124 (4.2%)	<0.001
Conjunctivitis (H10)	6427 (1.4%)	5534 (1.3%)	893 (3.3%)	<0.001
Gastroesophageal Reflux Disease (K21)	6805 (1.5%)	5570 (1.3%)	1235 (4.6%)	<0.001
Abnormalities of Breathing (R06)	5051 (1.1%)	3891 (0.9%)	1160 (4.3%)	<0.001
Back Pain/Low Back Pain (M54)	5539 (1.2%)	4566 (1.0%)	973 (3.6%)	<0.001
Dizziness and Giddiness (R42)	4665 (1.0%)	3894 (0.9%)	771 (2.9%)	<0.001
Joint Pain (M25)	4203 (0.9%)	3541 (0.8%)	662 (2.5%)	<0.001
Otitis Externa (H60)	4047 (0.9%)	3448 (0.8%)	599 (2.2%)	<0.001
Allergic Reaction (T78)	3207 (0.7%)	2524 (0.6%)	683 (2.5%)	<0.001
Cough (R05)	3202 (0.7%)	2589 (0.6%)	613 (2.3%)	<0.001
Urticaria (L50)	2906 (0.6%)	2320 (0.5%)	586 (2.2%)	<0.001
Psychiatric Disorder (F Codes)	2056 (0.4%)	1642 (0.4%)	414 (1.5%)	<0.001
Hypertension (I10)	2344 (0.5%)	1968 (0.4%)	376 (1.4%)	<0.001
**Cost Analysis (USD)**				
Laboratory Cost (USD), median (IQR)	0.00 (0.00 - 2.15)	0.00 (0.00 - 1.54)	0.00 (0.00 - 3.96)	<0.001
Radiology Cost (USD), median (IQR)	0.00 (0.00 - 2.43)	0.00 (0.00 - 2.52)	0.00 (0.00 - 2.17)	<0.001
Medication Order Cost (USD), median (IQR)	0.10 (0.00 - 0.91)	0.08 (0.00 - 0.84)	0.21 (0.00 - 1.09)	<0.001
Total Visit Cost (USD), median (IQR)	6.39 (3.33 - 21.88)	6.39 (3.33 - 21.73)	6.39 (3.38 - 22.55)	0.647

Notes: Values are presented as number (percentage) or median (IQR), as appropriate. Group comparisons were performed using the chi-square test for categorical variables and the Mann–Whitney U test for cost variables. A two-sided *p* value < 0.05 was considered statistically significant. A laboratory test was ordered in 29.3% of HFU vs 26.7% of non-HFU presentations, radiologic imaging in 40.5% vs 44.7%, and a medication order in 57.8% vs 51.4%; the remaining presentations incurred no cost of that type, which accounts for the median cost of 0.00 USD (a zero-inflated distribution)

Abbreviations: HFU, high-frequency user (≥4 ED visits within a 12-month period); USD, United States dollar; IQR, interquartile range

†Diagnostic categories are reported at the patient level: values are the number (%) of unique patients in whom the category was ever recorded at least once during the study period (HFU *n* = 26,871; non-HFU *n* = 440,013), so a patient may contribute to more than one category and column percentages do not sum to 100. Cost variables are reported at the visit level: values are the cost of a single ED presentation, and percentages and distributions for these rows are calculated against the total number of visits (726,696; 578,374; and 148,322 for the total, non-HFU and HFU groups, respectively), not the number of unique patients. Total visit cost is the sum of all charges billed for the presentation and includes the ED examination fee, procedures, consumables, observation and consultation charges in addition to the three components listed; it therefore exceeds their sum. Z20 (contact with and suspected exposure to communicable diseases) is reported here as a descriptive account of recorded diagnoses but was excluded from the regression models in Table 3, as it functions as an administrative, screening-related code

### Resource utilization and costs

Laboratory-related costs per ED presentation demonstrated statistically significant differences between groups (*p* < 0.001), despite identical median values. The median laboratory cost was 0.00 USD in both HFUs and non-HFUs; however, the interquartile range was wider among HFUs (0.00–3.96 USD) compared with non-HFUs (0.00–1.54 USD).

Radiology-related costs also differed significantly between groups (*p* < 0.001). While median radiology costs were 0.00 USD in both groups, cost dispersion was slightly greater among non-HFUs (IQR 0.00–2.52 USD) than among HFUs (IQR 0.00–2.17 USD).

Medication order–related costs were higher in the HFU group, with a median cost of 0.21 USD (IQR 0.00–1.09) compared with 0.08 USD (IQR 0.00–0.84) in non-HFUs (*p* < 0.001).

In contrast, total visit cost did not differ significantly between the groups. The median total cost per ED presentation was 6.39 USD in both HFUs (IQR 3.38–22.55) and non-HFUs (IQR 3.33–21.73), with no statistically significant difference observed (*p* = 0.647) (Table 2).

Resource-utilization rates were examined to determine whether the cost differences reflected more frequent ordering or the use of more expensive items. A higher proportion of HFU than non-HFU presentations involved any laboratory test (29.3% vs 26.7%) and any medication order (57.8% vs 51.4%), whereas any radiologic imaging was less frequent among HFUs (40.5% vs 44.7%) (all *p* < 0.001). Among presentations in which a given resource was used, the mean cost was similar between groups (laboratory 13.9 vs 12.8 USD; imaging 11.1 vs 11.8 USD; medication 5.0 vs 4.6 USD), indicating that the higher laboratory and medication costs among HFUs reflected more frequent ordering rather than the use of more expensive tests or treatments.

Because the very large number of presentations renders even trivial differences statistically significant, effect sizes were examined alongside the *p* values. For every cost component the effect size was negligible: the rank-biserial correlation (Cliff’s delta) was +0.03 for laboratory cost, −0.05 for radiology cost and +0.08 for medication cost, with common-language effect sizes close to 0.50 (0.51, 0.48 and 0.54, respectively). Thus, although laboratory, radiology and medication costs differed statistically, the differences were small in practical terms at the per-visit level and were attributable to the proportion of visits incurring any cost and to the upper tail of the distribution rather than to a shift in the median.

At the patient level, the resource burden was concentrated in the high-frequency group. Although cost per presentation was comparable between groups, HFUs accounted for 148,322 of 726,696 presentations (20.4%) while representing only 5.8% of patients; correspondingly, their cumulative annual laboratory, imaging and medication costs were approximately fourfold higher per patient (median 20.30 USD [IQR 5.31–61.60] vs 1.81 USD [IQR 0.23–12.53]; mean 63.2 vs 14.5 USD), so that this 5.8% subgroup accounted for 21.0% of these resource costs (Fig. 1).

### Factors associated with high-frequency emergency department use (patient level)

The regression analyses were performed at the patient level (*n* = 466,884 unique adult patients; 26,871 HFUs and 440,013 non-HFUs). All triage, shift, arrival-mode and diagnostic covariates are ever/history indicators denoting whether the characteristic was recorded at any visit during the study period. In univariable analyses, multiple demographic, visit-related and diagnostic variables were significantly associated with high-frequency ED use (Table 3).

**Table 3:** Univariable and multivariable logistic regression of factors associated with high-frequency emergency department use (patient level; *n* = 466,884 unique adult patients; 26,871 HFUs and 440,013 non-HFUs)

Variable	Univariate OR (95% CI)	p Value (Uni)	Multivariate aOR (95% CI)	p Value (Multi)
**Demographic Variables**				
Age (Per 1 year increase)	1.00 (1.00–1.00)	<0.001	1.00 (1.00–1.00)	0.014
Sex (male)	0.95 (0.93–0.97)	<0.001	1.16 (1.12–1.21)	<0.001
Ambulance Use (History)	1.91 (1.83–1.98)	<0.001	0.80 (0.70–0.92)	0.002
**Visit-Based Characteristics**				
Triage: Yellow Zone (History)	7.31 (7.06–7.57)	<0.001	4.19 (4.01–4.38)	<0.001
Triage: Red Zone (History)	2.28 (2.20–2.37)	<0.001	3.92 (3.45–4.46)	<0.001
Shift: Evening (History)	7.03 (6.78–7.29)	<0.001	3.72 (3.55–3.89)	<0.001
Shift: Night (History)	4.68 (4.57–4.80)	<0.001	2.75 (2.65–2.85)	<0.001
**Diagnosis Groups (ICD-10 Codes)**				
Soft Tissue Pain (M79)	7.75 (7.46–8.05)	<0.001	13.10 (12.48–13.74)	<0.001
Acute Upper Respiratory Infection (J06)	4.17 (4.06–4.27)	<0.001	7.01 (6.74–7.28)	<0.001
Unspecified Pain (R52)	4.92 (4.80–5.04)	<0.001	5.73 (5.52–5.94)	<0.001
Other Upper Respiratory Tract Diseases (J39)	4.19 (4.08–4.30)	<0.001	5.36 (5.15–5.58)	<0.001
Gastroenteritis and Colitis (K52)	3.71 (3.57–3.84)	<0.001	3.61 (3.40–3.83)	<0.001
Abdominal and Pelvic Pain (R10)	4.10 (3.94–4.26)	<0.001	5.21 (4.88–5.56)	<0.001
Malaise and Fatigue (R53)	4.74 (4.56–4.92)	<0.001	5.47 (5.13–5.83)	<0.001
General Examination Without Complaint (Z00)	4.64 (4.44–4.86)	<0.001	6.07 (5.62–6.55)	<0.001
Cystitis/Urinary Tract Infection (N30)	4.21 (4.01–4.42)	<0.001	5.53 (5.11–5.99)	<0.001
Nausea and Vomiting (R11)	4.35 (4.14–4.56)	<0.001	3.80 (3.50–4.12)	<0.001
Headache (R51)	4.13 (3.89–4.39)	<0.001	5.43 (4.91–6.00)	<0.001
Throat and Chest Pain (R07)	3.24 (3.03–3.46)	<0.001	4.46 (4.01–4.97)	<0.001
Conjunctivitis (H10)	2.70 (2.51–2.90)	<0.001	5.24 (4.68–5.87)	<0.001
Gastroesophageal Reflux Disease (K21)	3.76 (3.53–4.00)	<0.001	1.79 (1.61–2.00)	<0.001
Dyspnea/Shortness of Breath (R06)	5.06 (4.73–5.41)	<0.001	7.24 (6.43–8.14)	<0.001
Back Pain/Low Back Pain (M54)	3.58 (3.34–3.84)	<0.001	6.74 (6.01–7.55)	<0.001
Dizziness/Vertigo (R42)	3.31 (3.06–3.58)	<0.001	4.37 (3.83–4.99)	<0.001
Joint Pain (M25)	3.11 (2.86–3.39)	<0.001	5.55 (4.87–6.33)	<0.001
Otitis Externa (H60)	2.89 (2.64–3.15)	<0.001	5.29 (4.59–6.11)	<0.001
Allergic Reaction (T78)	4.52 (4.15–4.93)	<0.001	6.93 (5.99–8.02)	<0.001
Cough (R05)	3.95 (3.61–4.31)	<0.001	2.45 (2.10–2.86)	<0.001
Urticaria (L50)	4.21 (3.84–4.61)	<0.001	5.14 (4.40–6.01)	<0.001
Psychiatric Disorder (F Codes)	4.18 (3.75–4.66)	<0.001	4.41 (3.68–5.29)	<0.001
Hypertension (I10)	3.16 (2.83–3.53)	<0.001	4.57 (3.79–5.51)	<0.001

Notes: Analyses were performed at the patient level (one observation per unique patient; *n* = 466,884). All triage, shift, ambulance-arrival and ICD-10 diagnostic covariates are patient-level ever/history indicators denoting whether the characteristic was recorded at any visit during the 12-month study period; age is in years at first presentation and sex is a fixed characteristic. Because patients with a greater number of presentations had more opportunities to satisfy these conditions, the estimates describe factors associated with HFU status and must not be interpreted as causal or validated predictive effects. Multivariable models were adjusted for age, sex and the covariates listed. The ICD-10 code Z20 (contact with and suspected exposure to communicable diseases) was excluded from both the univariable and the multivariable models as an administrative, screening-related code. Model performance: apparent area under the ROC curve 0.95 within the study dataset (Fig. 2); neither internal validation by resampling nor external validation was performed. All tests were two-sided, and *p* values < 0.05 were considered statistically significant

Abbreviations: HFU, high-frequency user (≥4 ED visits within a 12-month period); OR, odds ratio; aOR, adjusted odds ratio; CI, confidence interval; Uni, univariable analysis; Multi, multivariable analysis

In the multivariable logistic regression model, several factors remained independently associated with high-frequency ED use. Male sex was associated with HFU status after adjustment (adjusted odds ratio [aOR] 1.16; 95% CI 1.12–1.21; *p* < 0.001); notably, the univariable analysis showed no male predominance, consistent with the near-equal sex distribution in Table 1, indicating that this association emerged only after adjustment for age, shift, triage and diagnostic covariates. Age showed a statistically significant but negligible association (aOR 1.00 per year; 95% CI 1.00–1.00; *p* = 0.014).

Although a history of ambulance use was positively associated with high-frequency ED use in univariable analysis, it was associated with lower odds of high-frequency use in the multivariable model (aOR 0.80; 95% CI 0.70–0.92; *p* = 0.002).

Aggregated visit characteristics showed the strongest associations. A history of yellow-triage presentation was associated with increased odds of high-frequency ED use (aOR 4.19; 95% CI 4.01–4.38; *p* < 0.001), as was a history of red-triage presentation (aOR 3.92; 95% CI 3.45–4.46; *p* < 0.001). Presentation timing was also associated with high-frequency ED use, with a history of evening-shift presentation showing the strongest association (aOR 3.72; 95% CI 3.55–3.89; *p* < 0.001), followed by a history of night-shift presentation (aOR 2.75; 95% CI 2.65–2.85; *p* < 0.001).

Multiple diagnostic categories were also associated with high-frequency ED use after multivariable adjustment. The strongest was soft tissue pain (M79; aOR 13.1; 95% CI 12.5–13.7; *p* < 0.001), followed by other symptom-based and non-specific groups, including acute upper respiratory infections (J06), unspecified pain (R52), other upper respiratory tract diseases (J39), gastroenteritis and colitis (K52), abdominal and pelvic pain (R10), malaise and fatigue (R53), general examination without complaint (Z00), urinary tract infection (N30), nausea and vomiting (R11) and headache (R51), together with additional musculoskeletal, allergic, ear–nose–throat and respiratory presentations as well as psychiatric (F-code) and cardiovascular (hypertension, I10) conditions; all remained associated with high-frequency ED use (all *p* < 0.001). The ICD-10 code Z20 was excluded from both models, and no Z20 term is present in Table 3.

**Fig. 2:**
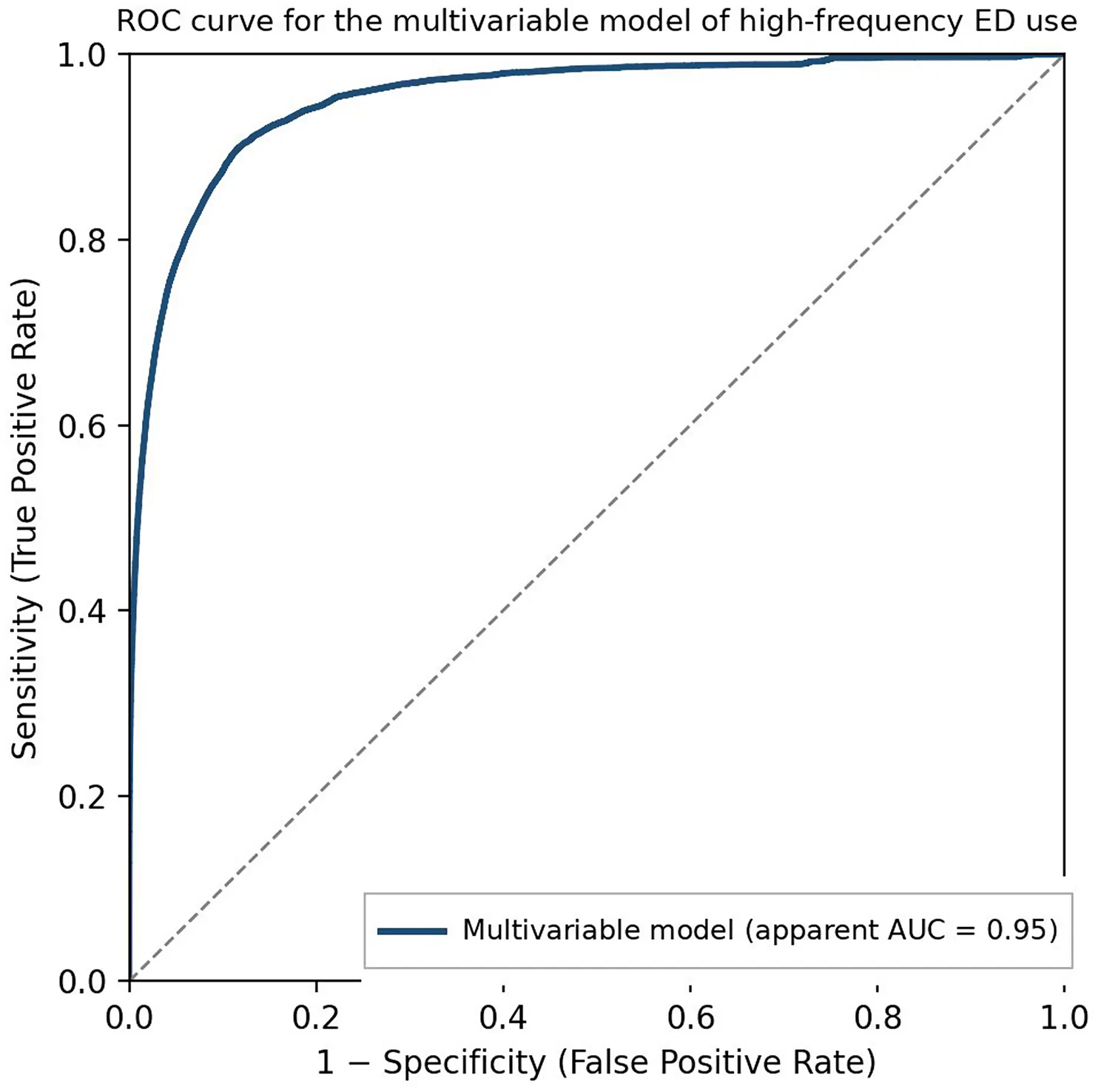
Receiver operating characteristic (ROC) curve for the patient-level multivariable logistic regression model of high-frequency emergency department use (*n* = 466,884 unique adult patients). The area under the curve (0.95) represents the apparent discrimination of the model within the study dataset; the model was neither internally validated by resampling nor externally validated, and this value is therefore likely to be optimistic. The ICD-10 code Z20 was excluded from the model

The apparent area under the ROC curve for the multivariable model was 0.95 within the study dataset (Fig. 2). Because every covariate is an ever/history indicator, patients with a greater number of presentations had more opportunities to meet these conditions, and the model was neither internally validated by resampling nor externally validated. This value therefore describes how well the model separates HFUs from non-HFUs in the data in which it was fitted, and it is likely to be optimistic; it does not establish validated predictive performance.

## Discussion

Emergency department (ED) utilization has increased worldwide, reflecting not only population growth but also structural changes in healthcare systems. In the United States, adult ED use rose from 17% in 1999 to 21.7% in 2019 [1]. Similar trends have been reported across diverse health systems, where increasing demand for emergency care has outpaced the expansion of accessible primary and out-of-hours services. Factors such as ease of access, limited continuity of care and restricted availability of primary care outside regular working hours have been consistently implicated.

Within this context, the proportion of HFUs observed in our study (5.8%) aligns with prevalence ranges reported in prior studies using commonly applied thresholds (≥4 annual ED visits) [4–6]. However, the clinical and operational relevance of HFUs lies not in their prevalence but in the disproportionate burden of utilization associated with them. Large population-based analyses have demonstrated that HFUs account for a relatively small proportion of patients while contributing to a substantial share of total ED visits, with utilization growth exceeding overall ED expansion [7]. Our findings similarly demonstrate marked clustering of ED use within a limited subgroup.

HFUs in our cohort were marginally younger than non-HFUs, suggesting that high-frequency ED use is not restricted to older adults with multimorbidity. Prior studies have shown that younger adults may use EDs as substitutes for primary care, particularly outside regular working hours [14]. In our data, HFUs presented more often at night at the visit level, and out-of-hours presentation histories (evening and night shifts) remained associated with HFU status after adjustment, which is consistent with a contribution of access barriers beyond standard daytime services.

Triage patterns further indicate that high-frequency attendance cannot be explained solely by non-urgent use. Although green-triage presentations were less common among HFUs, symptom-based and pain-related diagnoses predominated. Similar diagnostic profiles have been described previously, supporting the concept that HFUs often present with recurrent, nonspecific complaints rather than discrete acute conditions [5, 15]. Together, these findings reinforce the heterogeneity of HFUs and suggest the presence of distinct utilization phenotypes. The higher proportion of yellow-triage and lower proportion of green-triage presentations among HFUs (Table 1) further indicate that their attendances were not uniformly low-acuity. This pattern may reflect acute exacerbations of recurrent or poorly controlled conditions, symptom amplification during repeated presentations, or differences in triage assignment among frequently attending patients; the present data cannot distinguish these explanations, which therefore remain hypotheses for future study.

Better accessibility and continuity of primary care have been associated with lower ED use across diverse health systems; strengthening daytime and out-of-hours primary care access may therefore warrant evaluation as a means of mitigating the repeated, frequently out-of-hours attendance observed in this subgroup [16, 17].

Regional evidence from Türkiye is consistent with these access- and crowding-related dynamics. A recent single-center Turkish study reported that patients who leave the ED without being seen are both a consequence and a contributor to ED crowding, with older adults at particular risk of hospitalization on return visits [18]; and, among older adults who use the ED frequently, the interval between successive visits has been associated with mortality [19]. These local findings reinforce the relevance of crowding, access and repeated-visit patterns to the high-frequency use observed in our cohort.

The cost findings require careful framing. Total cost per visit was similar between HFUs and non-HFUs, and although the laboratory, radiology and medication cost comparisons reached statistical significance, the corresponding effect sizes were negligible (rank-biserial correlations of +0.03, −0.05 and +0.08, respectively, with common-language effect sizes near 0.50). The statistical significance therefore reflects the very large sample rather than economically meaningful per-visit differences, and the differences in individual cost components should be regarded as small in practical terms. The interpretable signal at the visit level is instead the higher proportion of presentations involving laboratory testing and medication orders among HFUs, which is consistent with more frequent ordering rather than the use of more expensive tests or treatments; this pattern accords with reports that growth in ED expenditure has been attributed largely to rising per-patient treatment intensity rather than to visit volume alone [2]. More fundamentally, the principal finding of this study is the cumulative, patient-level resource burden associated with repeated attendance: although cost per presentation was comparable, HFUs comprised 5.8% of patients yet accounted for approximately one-fifth of all presentations and of cumulative laboratory, imaging and medication costs, with cumulative per-patient resource costs roughly fourfold higher.

From a pharmaco-economic perspective, the higher pharmacologic treatment costs observed among HFUs are notable even though the per-visit difference was small. Recurrent medication use may reflect symptom persistence, pharmacologic ordering behavior in the ED setting, or the absence of effective longitudinal care pathways. These findings suggest that ED prescribing patterns may be influenced not only by clinical need but also by utilization behavior; this is a hypothesis generated by the observed pattern rather than a demonstrated mechanism, and it may warrant evaluation as a domain for resource stewardship.

The multivariable model identified male sex, a history of higher-acuity triage categories and a history of evening- and night-shift presentation as factors associated with HFU status. In contrast, a history of ambulance arrival—while positively associated in univariable analysis—was associated with lower odds of high-frequency ED use after adjustment, which may indicate that ambulance use reflects acuity-driven access rather than repeated attendance. Two cautions apply to all of these estimates. First, every covariate was defined as whether a patient had ever experienced a given triage level, shift, arrival mode or diagnosis during the study period, and patients with more visits necessarily had more opportunities to meet these conditions; the estimates therefore describe associations with HFU status and not causal or validated predictive effects. Second, the AUC of 0.95 describes the apparent discrimination of the model within the dataset in which it was fitted, without internal or external validation, and is likely to be optimistic. Collectively, these results are consistent with a contribution of system-level and structural determinants rather than patient behavior alone; however, because the dataset lacked information on socioeconomic status, insurance, comorbidity and chronic-disease burden, and primary care access, these access-related explanations should be regarded as hypotheses generated by the observed patterns rather than as established mechanisms [3].

The literature on managing high-frequency ED use consistently identifies three complementary intervention domains: (i) case management and individualized care plans, (ii) improved access to primary and out-of-hours care, and (iii) identification and mitigation of social determinants of health. Systematic reviews and randomized studies report that targeted case management can reduce ED utilization among HFUs and, in some settings, lower healthcare costs [20–23]. Improving access to primary care and out-of-hours services has been associated with reduced reliance on EDs by redirecting appropriate patients to alternative care pathways [24]. In addition, growing evidence highlights the influence of social determinants—such as housing instability, economic vulnerability and limited social support—on repeated ED use, supporting the evaluation of social risk screening and referral within ED workflows [25, 26]. The present study did not evaluate any intervention and therefore cannot indicate which, if any, of these approaches would be effective in this setting.

This study represents one of the largest single-center analyses of high-frequency ED use reported to date. By integrating utilization patterns, diagnostic profiles and disaggregated cost components within an internationally comparable framework, and by separating patient-level from visit-level findings throughout, it contributes to the global literature on HFUs and provides descriptive evidence relevant to health system planning. The observed concentration of utilization, the symptom-driven diagnostic pattern and the out-of-hours presentation profile together identify domains that may warrant further evaluation in the design of emergency care services.

## Limitations

This study has several limitations. Its retrospective, single-center design limits causal inference and generalizability, and all findings are associations observed within routinely collected administrative data.

First, the findings are contingent on the definition of an HFU as a patient with at least four ED visits per year. Although this is the most widely applied threshold, it is not universal, and alternative thresholds (e.g. ≥7 or ≥ 10 visits per year, used elsewhere to delineate “highly frequent” users or “super-utilizers”) were not examined. No sensitivity analysis using alternative thresholds was performed, and the size, composition and resource profile of the high-utilization subgroup would be expected to change under a different definition. The results should therefore be read as applying to the ≥4 visits/year definition specifically.

Second, data on socioeconomic status, health insurance status, comorbidity and chronic-disease burden, medication history outside the ED, and access to or use of primary care were not available in the dataset. These are among the strongest known correlates of repeated ED attendance, and their absence means that the access-related and system-level explanations discussed above could not be tested and remain hypotheses rather than findings. Residual confounding by these unmeasured factors cannot be excluded for any of the reported associations.

Third, all regression covariates were patient-level ever/history indicators, so that patients with more presentations had more opportunities to satisfy each condition. The reported odds ratios describe factors associated with HFU status and must not be interpreted as causal effects, and the apparent AUC of 0.95, obtained without internal or external validation, is likely to be optimistic and does not represent validated predictive performance.

Fourth, cost analyses were limited to direct ED-related charges billed under the national reimbursement tariff and did not include downstream inpatient or outpatient costs, indirect costs, or societal costs; the absolute values are therefore not comparable to commercial or list charges from other health systems. Fifth, diagnostic classifications relied on routinely entered ICD-10 codes and may be subject to misclassification, and administrative codes such as Z20 illustrate the limits of using coded diagnoses as clinical descriptors. Finally, exclusion of 62,994 records (8.0% of the raw extract), principally for age under 18 years and for missing or invalid identifiers precluding patient linkage, may have introduced selection bias, and counts for the individual exclusion reasons were not retained separately.

For these reasons, the recommendations discussed above are presented as areas for future evaluation rather than as interventions shown by this study to be effective. These findings suggest that out-of-hours care pathways and the use of diagnostic and prescribing resources may warrant further evaluation. However, this observational study cannot determine which interventions would reduce frequent ED use; prospective studies linking utilization data to socioeconomic, comorbidity and primary-care variables, and trials of specific care pathways, would be required for that purpose.

## Conclusions

HFUs accounted for a small fraction of adult ED patients yet were associated with a disproportionate share of emergency care utilization. In this large urban cohort, HFUs represented 5.8% of adult patients but accounted for 20.4% of ED presentations and approximately one-fifth of cumulative laboratory, imaging and medication costs. Total cost per visit was similar between the groups, and differences in individual cost components were small in practical terms; the principal finding is therefore the cumulative resource burden associated with repeated ED attendance rather than a higher cost of the individual encounter. High-frequency attendance was associated with symptom-based presentations, a history of higher-acuity triage categories and repeated out-of-hours access. These findings suggest that out-of-hours care pathways and the use of diagnostic and prescribing resources may warrant further evaluation at the system level. However, this observational study cannot determine which interventions would reduce frequent ED use, and its findings should be interpreted as associations that generate hypotheses for prospective evaluation.

## Data Availability

The datasets generated and/or analyzed during the current study are not publicly available because they consist of routinely collected hospital administrative and clinical records that remain the property of Başakşehir Çam and Sakura City Hospital, and because the ethics approval obtained for this work (Clinical Research Ethics Committee No. 1, protocol/decision number 2025–262, 27 August 2025) covers only the analyses reported here and does not authorize public deposition of the underlying patient-level records. De-identified data supporting the findings of this study are available from the corresponding author (Raif Kaan Bas, raifkaanbas@gmail.com) on reasonable request, subject to approval by the institutional review board and to any data-use agreement required by the hospital. All aggregate data underlying the reported results—patient and visit counts, proportions, cost distributions and regression estimates—are presented in full in the manuscript, its tables and its figures.
